# Exosomes as Carriers of Alzheimer's Amyloid-ß

**DOI:** 10.3389/fnins.2017.00229

**Published:** 2017-04-25

**Authors:** Kohei Yuyama, Yasuyuki Igarashi

**Affiliations:** Laboratory of Biomembrane and Biofunctional Chemistry, Graduate School of Advanced Life Science, Hokkaido UniversitySapporo, Japan

**Keywords:** exosome, Alzheimer's disease, amyloid-ß, glycosphingolipid, microglia

## Abstract

The intracerebral level of the aggregation-prone peptide, amyloid-ß (Aß), is constantly maintained by multiple clearance mechanisms, including several degradation enzymes, and brain efflux. Disruption of the clearance machinery and the resultant Aß accumulation gives rise to neurotoxic assemblies, leading to the pathogenesis of Alzheimer's disease (AD). In addition to the classic mechanisms of Aß clearance, the protein may be processed by secreted vesicles, although this possibility has not been extensively investigated. We showed that neuronal exosomes, a subtype of extracellular nanovesicles, enwrap, or trap Aß and transport it into microglia for degradation. Here, we review Aß sequestration and elimination by exosomes, and discuss how this clearance machinery might contribute to AD pathogenesis and how it might be exploited for effective AD therapy.

## Introduction

Exosomes are a class of extracellular membrane vesicles with uniform spherical shape and a diameter of 70–150 nm. They are generated and secreted via exocytosis of intraluminal vesicles (ILVs) of multivesicular bodies (MVBs) by most cell types, including those in the central nervous system (CNS). Cultured neurons and glial cells such as microglia, oligodendrocytes, and astrocytes release exosomes into the medium and cerebrospinal fluid (CSF) of humans and model species such as mice and monkeys (Yuyama et al., 2015). *In vitro* studies have revealed that exosomes are involved in multiple physiological CNS processes, including the formation of the myelin sheath and regeneration of damaged axons (Bakhti et al., 2011; Lopez-Verrilli et al., 2013; Yuyama and Igarashi, 2016). On the other hand, because exosomes contain the proteins related to neurological diseases, such as prion disease, Parkinson's disease, and amyotrophic lateral sclerosis (ALS), and transport them among cells, the connection of CNS exosomes to the pathogenesis and progression of these diseases has attracted a great deal of attention (Howitt and Hill, 2016). Exosomes also contain two major pathological factors of Alzheimer's disease (AD), amyloid-ß protein (Aß) and tau, and the commitment of CNS exosomes to pathogenesis of the disease is under investigation. Our recent studies suggest that neuron-derived exosomes may participate in Aß clearance in the brains. In this short review, we describe the pathway of Aß clearance by the exosomes and discuss the potential significance of novel therapeutic and prevention strategies of AD in use of the exosomal functions.

## Amyloid-ß in Alzheimer's disease

AD, a neurological disorder associated with irreversible and progressive loss of memory and cognitive functions, is pathologically characterized by intracerebral deposits of Aß amyloid called senile plaques. Aß, which accumulates gradually over a long period of time (>15 years) prior to symptomatic onset, elicits other pathological hallmarks, such as neurofibrillary tangles (NFTs) and neuronal loss, that are directly linked to symptoms of the disease (Hardy and Selkoe, 2002). Aß is a ~4 kDa peptide generated by physiological processing of amyloid precursor protein (APP), a membrane protein composed of 695–770 amino acids in length. In neurons, the amyloidogenic processing of APP is performed by two proteases, ß- and γ-secretases. ß-secretase activity is mediated by a type I membrane protein called ß-site APP-cleaving enzyme 1 (BACE1), whereas γ-secretase cleavage is mediated by an intramembrane protease complex containing presenilin, witch harbors the catalytic domain. APP is transported to the plasma membranes and subsequently internalized in endocytic pathways. Both secretases are also sorted into endocytic compartments that maintain the optimal pH; hence Aß is primarily generated in the endocytic pathway (Rajendran and Annaert, 2012).

Steady-state levels of brain Aß are controlled by the balance between generation and degradation/clearance. In the case of familial AD, genetic alterations of molecules involved in generating Aß such as APP and presenilin, appear to increase Aß assembly by promoting production of aggregate-prone Aß. In sporadic AD, a common form of AD, the rate of Aß elimination in the brain is reduced (Mawuenyega et al., 2010). The slower clearance indicates perturbation of Aß clearance, for example, via a decrease in catabolism due to reduced proteolysis or impaired efflux across the blood-brain barrier. However, the precise mechanism of Aß amyloid accumulation remains controversial; a number of other events that may influence the deposit, such as endocytic perturbations, Aß seed formation and diabetic states, have been found in AD brains (Cataldo et al., 1997; Ariga et al., 2008; Ashraf et al., 2014; Danish Rizvi et al., 2015).

## Association of Aß on exosomes

In some neurons, endocytic perturbations such as endosome enlargement occur at the early stage of AD, when Aß levels begin to rise (Cataldo et al., 1997, 2001). Intracellular Aß accumulates in the abnormal endosomes, suggesting these bodies contribute to the earliest elevation of Aß (Cataldo et al., 2004). Electron microscopic observations of the brains of APP/PS1 transgenic mice and AD patients reveal that the neuronal endosomes containing accumulated Aß include MVBs, whose ILVs are precursors of exosomes (Takahashi et al., 2002; Langui et al., 2010). In 2006, Rajendran and co-workers grasped and reported that a minute fraction of Aß is released in association with exosomes from APP-transfected cells to the culture medium (Rajendran et al., 2006). In addition, they have showed that Alix, an exosomal marker protein, is enriched around human amyloid plaques, suggesting that Aß associated with exosomes contributes to plaque formation. Aß is detected also in the exosomes derived from the CSF collected from APP transgenic mice and cynomolgus monkeys (Yuyama et al., 2015). As well as Aß, APP, and its metabolites, APP C-terminal fragment (CTF), and APP intercellular domain (AICD), are present in exosomes and released to the extracellular space (Vingtdeux et al., 2007; Sharples et al., 2008; Perez-Gonzalez et al., 2012). In human, a subset of blood exosomes contain Aß, and its levels are elevated in MCI and AD patients (Fiandaca et al., 2015; see section Exosomes as Tools for AD Therapy).

Aß is produced via sequential processing of APP by the two secretases described above, is translocated into intraluminal space of endosomal compartment such as MVB and then released from the cell via the recycling pathway. Hence when Aß is present in exosomes, it is likely topologically bound to the surface membranes. Immunoelectron microscopy reveals that Aß is attached to the surface of the exosomes derived from APP-expressed N2a cells (Rajendran et al., 2006). Surface plasmon resonance analysis performed by our group also shows that N2a-derived exosomes injected onto the immobilized synthetic Aß (Aß_1−40_, Aß_1−42_, and Aß_1−38_) exhibit significantly elevated resonance signals, demonstrating that the exosome interacts with the Aßs (Yuyama et al., 2014).

What, then, molecular mechanism responsible for the binding of Aß to exosomes? Aß binds to glycosphingolipids (GSLs), a group of glycan-linked membrane lipids (Yanagisawa et al., 1995; Ariga et al., 2008). GSLs are localized on the outer layer of cellular and exosome membranes, and their glycans are exposed to the external milieu. GSLs move laterally across the membranes and form clusters at high densities: monomeric Aß recognizes the GSL clusters and bind to them (Yamamoto et al., 2008). GSLs also accelerate Aß assembly into fibrils. Assemblies of Aß with GM1, a sialic acid–linked GSL, were recovered from the tissues and interstitial fluid of brains of aged monkeys and AD patients (Langui et al., 2010; Hong et al., 2014). Our quantitative GSL-glycomic analysis of N2a-derived exosomes and their parental cells revealed that GSLs are more abundant in exosomes than cells (Yuyama et al., 2014), and that sialylated GSLs such as GM1 are particularly concentrated in the exosomes. Degradation of GSL-glycans or sialic acids by endoglycoceramidase (EGCase) or sialidase efficiently prevents the association between Aß and exosomes (Yuyama et al., 2014). An *in vivo* study has revealed that N2a-derived exosomes, which injected into the hippocampus of APP transgenic mice, also associate with endogenous Aß. Removal of GSL-glycans on neuronal exosomes by EGCase abolishes the Aß-binding activity of the exosomes.

We also collected the exosomes from primary cultures of mouse neurons, astrocytes and microglia, and analyzed the profiles of their GSL-derived glycans, demonstrating that significantly more GSLs are present in exosomes from neurons than in those from glial cells (Yuyama et al., 2015). Accordingly, only neuronal exosomes, but not glial exosomes, are associated with Aß. These observations indicate that Aß can associate with accumulated and clustered GLSs on exosomal membranes.

In addition to GSLs, neuronal exosomes bind with Aß through prion protein (PrP) (An et al., 2013). PrP is a glycosylphosphatidylinositol (GPI)-anchored protein and localized on the outer leaflet of the membranes of both neuronal cells and exosomes, and can function as a receptor for Aß oligomeric species known as Aß-derived diffusible ligands (ADDLs). Binding of ADDLs to cellular PrP induces toxic signaling and disrupts synaptic plasticity (Laurén et al., 2009). In a study in rat in which exogenously prepared ADDLs are infused into the hippocampus, co-administration of exosomes derived from neuronal cultures or human CSF neutralizes the ADDLs-induced impairment of long-term potentiation. Thus, exosomal PrP may trap ADDLs to prevent their cellular toxicity.

## Exosome-dependent Aß clearance

Brain-resident phagocytes microglia can participate in internalization of the exosomes derived from various types of cells (Fitzner et al., 2011; Yuyama et al., 2012). When exosomes are added to mixed brain cultures containing all major cell types, they are preferentially uptaken by microglia (Fitzner et al., 2011). An *in vivo* study of mouse brains reveals that cultured neuron-derived exosomes injected intracerebrally are almost exclusively internalized into microglia (Yuyama et al., 2014). Membrane lipid phosphatidylserine (PS) is localized in the inner leaflet of the plasma membrane via an energy-requiring mechanism. Apoptotic cells rapidly lose the asymmetric localization of PS, and are phagocytized and cleared by microglia; this process is mediated by recognition of PS exposed on the cell surface by the microglial PS receptor (Schlegel and Williamson, 2001). Notably, exosomal PS is localized in the outer leaflet of the surface membranes, as well as apoptotic cells, and can be recognized by the microglial PS receptor (Miyanishi et al., 2007). Therefore, when annexin V, a PS-binding toxin, is exposed to exosomes to mask the surface PS, the phagocytosis of neuron-derived exosomes into microglial cells is significantly inhibited (Yuyama et al., 2012).

**Figure 1:**
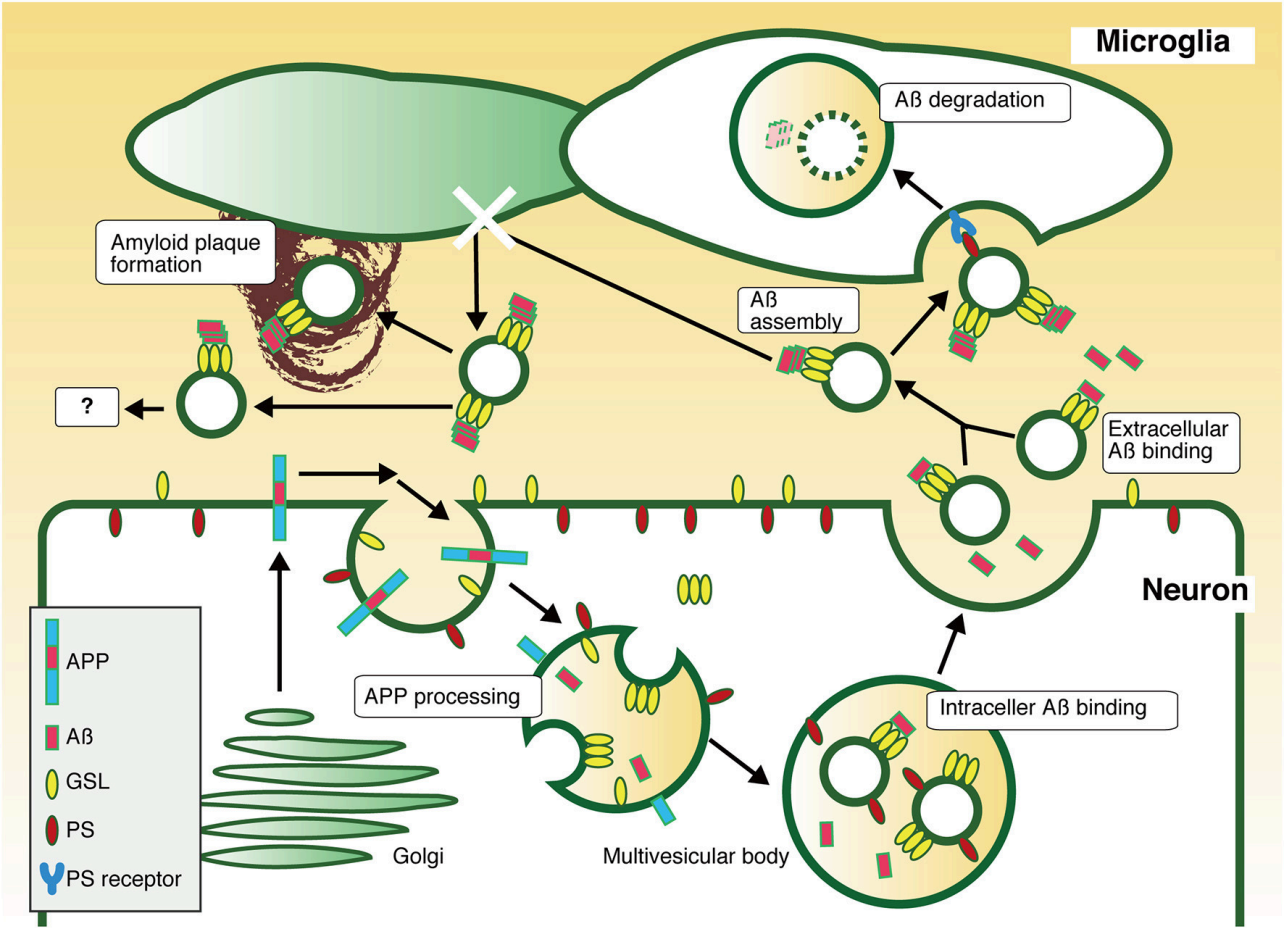
**Pathway of exosome-dependent Aß clearance**. APP is sorted into endosomes with acidic pH, where it is sequentially cleaved by secretases to produce Aß. The resultant Aß is released to the extracellular milieu through fusion of recycling endosomes or MVBs with the plasma membrane. Some Aß is associated with exosomes in MVBs or in the extracellular space, an interaction mediated by GSLs. Exosomes stack Aß on their surface by promoting the formation of nontoxic Aß assembly by GSLs, followed by incorporation of Aß fibrils into microglia in a PS-dependent manner, resulting in degradation. Thus, neuronal exosomes are likely to promote Aß clearance. In absence of microglial phagocytic activity, exosome-associated Aß might induce any pathogenic event, such as amyloid plaque formation.

The exosomes associated with Aß are also internalized into microglia as well as native exosomes. The exosome-bound Aß is transported through the endocytic pathway to microglial lysosomes, where it is degraded. Thus, neuronal exosomes, which can trap Aß, act as couriers of Aß for Aß clearance. In co-cultures of human APP-transfected N2a cells and microglial BV2 cells, promotion of exosome generation in N2a by knockdown of sphingomyelin synthase increases engulfment of extracellular Aß by BV2 and decreases the levels of Aß in the culture medium. Although, free Aß can be taken up by microglia for degrade, the exosomes might clear Aß more efficiently by transporting it in assembled forms collectively (Figure 1).

To determine whether exosomes affect the levels of brain Aß, we also performed intracerebrally infusion of neuronal exosomes into mouse brains (Yuyama et al., 2014). Continuous infusion of neuronal exosomes for 2 weeks into 4-month-old APP transgenic mice using an osmotic mini-pump decreases Aß levels and attenuates the reduction in synaptic densities induced by Aß toxicity in the hippocampus. Likewise, the infusion into the brains of 12-month-old mice also decreases Aß amyloid depositions (Yuyama et al., 2015). These studies clearly demonstrated that intracerebral exosome administration ameliorates Aß-related AD pathogenesis. Therefore, improvement of Aß clearance to prevent its toxicity by exosome supplementation or promotion of exosome generation might provide a novel therapeutic approach for AD.

## Exosomes as tools for AD therapy

Acceleration of Aß clearance by administration of neuronal exosomes or promotion of exosome production represents a novel therapeutic approach for AD. Instead of natural exosomes, exosome-like synthetic liposomes that contain GSLs responsible for capturing Aß and PS for the purpose of glial internalization would have several advantages, including uniformity and the absence of contaminants. Notably, the Aß-degrading enzymes insulin-degrading enzyme and neprilysin are present in the exosomes released from microglia and adipose tissue-derived mesenchymal stem cells, respectively (Bulloj et al., 2010; Katsuda et al., 2013). The exosomes have also been studied as a delivery platform, encapsulating reagents or siRNAs (Alvarez-Erviti et al., 2011; Zhuang et al., 2011). Peripheral injection of exosomes containing BACE1 siRNA or curcumin can be targeted into the brain to ameliorate AD-like pathology in mice. As nanotechnological approaches, these functional exosomes or exosome-like liposomes, or even fusion vesicles of both types (Ashraf et al., 2015; Sato et al., 2016; Ansari et al., 2017) that restore brain capacity, might be a valuable tools for AD therapy.

## Conclusions

In this review, we summarize the roles of exosomes in AD by focusing on the potential beneficial effects in Aß degradation/clearance. Improvement of Aß clearance by the regulation of the exosome production or intracerebral administration of the exosomes may be a potent strategy for AD therapy. However, recent findings implicate that exosomes act as double-edged sword in AD. The exosomes derived from Aß-stimulated astrocytes and aggregated tau-treated microglia are involved in Aß aggregation and tau interneuronal propagation, respectively (Asai et al., 2015; Dinkins et al., 2016; Xiao et al., 2017). Depending on the cell origin and the pathological stage of the disease, exosomes may have detrimental roles contributing to worsening or spread of the pathogenesis. Further deliberate researches on the pathophysiological properties of these vesicles would open the door to develop new therapeutic strategies for AD.

## Author contributions

KY Participate in drafting the article. YI Participate in critically revising the article for important intellectual content.

### Conflict of interest statement

The authors declare that the research was conducted in the absence of any commercial or financial relationships that could be construed as a potential conflict of interest.

## References

[B1] Alvarez-Erviti L. Seow Y. Yin H. Betts C. Lakhal S. Wood M. J. (2011). Delivery of siRNA to the mouse brain by systemic injection of targeted exosomes. Nat. Biotechnol. 29, 341–345. 10.1038/nbt.1807 21423189

[B2] An K. Klyubin I. Kim Y. Jung J. H. Mably A. J. O'Dowd S. T.. (2013). Exosomes neutralize synaptic-plasticity-disrupting activity of Aβ assemblies *in vivo*. Mol. Brain 6:47. 10.1186/1756-6606-6-47 24284042 PMC4222117

[B3] Ansari S. A. Satar R. Perveen A. Ashraf G. M. (2017). Current opinion in Alzheimer's disease therapy by nanotechnology-based approaches. Curr. Opin. Psychiatry 30, 128–135. 10.1097/YCO.0000000000000310 28009724

[B4] Ariga T. McDonald M. P. Yu R. K. (2008). Role of ganglioside metabolism in the pathogenesis of Alzheimer's disease–a review. J. Lipid Res. 49, 1157–1175. 10.1194/jlr.R800007-JLR200 18334715 PMC2386904

[B5] Asai H. Ikezu S. Tsunoda S. Medalla M. Luebke J. Hayder T.. (2015). Depletion of microglia and inhibition of exosome synthesis halt tau propagation. Nat. Neurosci. 18, 1584–1593. 10.1038/nn.4132 26436904 PMC4694577

[B6] Ashraf G. M. Greig N. H. Khan T. A. Hassan I. Tabrez S. Shakil S.. (2014). Protein misfolding and aggregation in Alzheimer's disease and type 2 diabetes mellitus. CNS Neurol. Disord. Drug Targets 13, 1280–1293. 10.2174/1871527313666140917095514 25230234 PMC5193501

[B7] Ashraf G. M. Tabrez S. Jabir N. R. Firoz C. K. Ahmad S. Hassan I.. (2015). An overview on global trends in nanotechnological approaches for alzheimer therapy. Curr. Drug Metab. 16, 719–727. 10.2174/138920021608151107125757 26560324

[B8] Bakhti M. Winter C. Simons M. (2011). Inhibition of myelin membrane sheath formation by oligodendrocyte-derived exosome-like vesicles. J. Biol. Chem. 286, 787–796. 10.1074/jbc.M110.190009 20978131 PMC3013037

[B9] Bulloj A. Leal M. C. Xu H. Castaño E. M. Morelli L. (2010). Insulin-degrading enzyme sorting in exosomes: a secretory pathway for a key brain amyloid-β degrading protease. J. Alzheimer's Dis. 19, 79–95. 10.3233/JAD-2010-1206 20061628 PMC4414343

[B10] Cataldo A. M. Barnett J. L. Pieroni C. Nixon R. A. (1997). Increased neuronal endocytosis and protease delivery to early endosomes in sporadic alzheimer's disease: neuropathologic evidence for a mechanism of increased β-amyloidogenesis. J. Neurosci. 17, 6142–6151. 9236226 10.1523/JNEUROSCI.17-16-06142.1997 PMC6568334

[B11] Cataldo A. M. Petanceska S. Terio N. B. Peterhoff C. M. Durham R. Mercken M.. (2004). Aβ localization in abnormal endosomes: association with earliest Aβ elevations in AD and down syndrome. Neurobiol. Aging 25, 1263–1272. 10.1016/j.neurobiolaging.2004.02.027 15465622

[B12] Cataldo A. Rebeck G. W. Ghetri B. Hulette C. Lippa C. Van Broeckhoven C.. (2001). Endocytic disturbances distinguish among subtypes of Alzheimer's disease and related disorders. Ann. Neurol. 50, 661–665. 11706973

[B13] Danish Rizvi S. M. Shaikh S. Waseem S. M. Shakil S. Abuzenadah A. M. Biswas D. (2015). Role of anti-diabetic drugs as therapeutic agents in Alzheimer's disease. EXCLI J. 14, 684–696. 10.17179/excli2015-252 27152105 PMC4849108

[B14] Dinkins M. B. Enasko J. Hernandez C. Wang G. Kong J. Helwa I.. (2016). Neutral sphingomyelinase-2 deficiency ameliorates Alzheimer's disease pathology and improves cognition in the 5XFAD Mouse. J. Neurosci. 36, 8653–8667. 10.1523/JNEUROSCI.1429-16.2016 27535912 PMC4987436

[B15] Fiandaca M. S. Kapogiannis D. Mapstone M. Boxer A. Eitan E. Schwartz J. B.. (2015). Identification of preclinical Alzheimer's disease by a profile of pathogenic proteins in neurally derived blood exosomes: a case-control study. Alzheimers Dement. 11, 600–607. 10.1016/j.jalz.2014.06.008 25130657 PMC4329112

[B16] Fitzner D. Schnaars M. van Rossum D. Krishnamoorthy G. Dibaj P. Bakhti M.. (2011). Selective transfer of exosomes from oligodendrocytes to microglia by macropinocytosis. J. Cell Sci. 124, 447–458. 10.1242/jcs.074088 21242314

[B17] Hardy J. Selkoe D. J. (2002). The amyloid hypothesis of alzheimer's disease: progress and problems on the road to therapeutics. Science 297, 353–356. 10.1126/science.1072994 12130773

[B18] Hong S. Ostaszewski B. L. Yang T. O'Malley T. T. Jin M. Yanagisawa K.. (2014). Soluble Aβ oligomers are rapidly sequestered from brain ISF *in vivo* and bind GM1 ganglioside on cellular membranes. Neuron 82, 308–319. 10.1016/j.neuron.2014.02.027 24685176 PMC4129520

[B19] Howitt J. Hill A. F. (2016). Exosomes in the pathology of neurodegenerative diseases. J. Biol. Chem. 291, 26589–26597. 10.1074/jbc.R116.757955 27852825 PMC5207170

[B20] Katsuda T. Tsuchiya R. Kosaka N. Yoshioka Y. Takagaki K. Oki K.. (2013). Human adipose tissue-derived mesenchymal stem cells secrete functional neprilysin-bound exosomes. Sci. Rep. 3:1197. 10.1038/srep01197 23378928 PMC3561625

[B21] Langui D. Girardot N. El Hachimi K. H. Allinquant B. Blanchard V. Pradier L.. (2010). Subcellular topography of neuronal Aβ peptide in APPxPS1 transgenic mice. Am. J. Pathol. 165, 1465–1477. 10.1016/S0002-9440(10)63405-0 15509518 PMC1618656

[B22] Laurén J. Gimbel D. A. Nygaard H. B. Gilbert J. W. Strittmatter S. M. (2009). Cellular prion protein mediates impairment of synaptic plasticity by amyloid-β oligomers. Nature 457, 1128–1132. 10.1038/nature07761 19242475 PMC2748841

[B23] Lopez-Verrilli M. A. Picou F. Court F. A. (2013). Schwann cell-derived exosomes enhance axonal regeneration in the peripheral nervous system. Glia 61, 1795–1806. 10.1002/glia.22558 24038411

[B24] Mawuenyega K. G. Sigurdson W. Ovod V. Munsell L. Kasten T. Morris J. C.. (2010). Decreased clearance of CNS β-Amyloid in Alzheimer's disease. Science 330:1774. 10.1126/science.1197623 21148344 PMC3073454

[B25] Miyanishi M. Tada K. Koike M. Uchiyama Y. Kitamura T. Nagata S. (2007). Identification of Tim4 as a phosphatidylserine receptor. Nature 450, 435–439. 10.1038/nature06307 17960135

[B26] Perez-Gonzalez R. Gauthier S. A. Kumar A. Levy E. (2012). The exosome secretory pathway transports amyloid precursor protein carboxyl-terminal fragments from the cell into the brain extracellular space. J. Biol. Chem. 287, 43108–43115. 10.1074/jbc.M112.404467 23129776 PMC3522305

[B27] Rajendran L. Annaert W. (2012). Membrane trafficking pathways in Alzheimer's disease. Traffic 13, 759–770. 10.1111/j.1600-0854.2012.01332.x 22269004

[B28] Rajendran L. Honsho M. Zahn T. R. Keller P. Geiger K. D. Verkade P.. (2006). Alzheimer's disease β-Amyloid peptides are released in association with exosomes. Proc. Natl. Acad. Sci. U.S.A. 103, 11172–11177. 10.1073/pnas.0603838103 16837572 PMC1544060

[B29] Sato Y. T. Umezaki K. Sawada S. Mukai S. A. Sasaki Y. Harada N.. (2016). Engineering hybrid exosomes by membrane fusion with liposomes. Sci. Rep. 6:21933. 10.1038/srep21933 26911358 PMC4766490

[B30] Schlegel R. A. Williamson P. (2001). Phosphatidylserine, a death knell. Cell Death Differ. 8, 551–563. 10.1038/sj.cdd.4400817 11536005

[B31] Sharples R. A. Vella L. J. Nisbet R. M. Naylor R. Perez K. Barnham K. J.. (2008). Inhibition of γ-secretase causes increased secretion of amyloid precursor protein C-terminal fragments in association with exosomes. FASEB J. 22, 1469–1478. 10.1096/fj.07-9357com 18171695

[B32] Takahashi R. H. Milner T. A. Li F. Nam E. E. Edgar M. A. Yamaguchi H.. (2002). Intraneuronal Alzheimer Aβ42 accumulates in multivesicular bodies and is associated with synaptic pathology. Am. J. Pathol. 161, 1869–1879. 10.1016/S0002-9440(10)64463-X 12414533 PMC1850783

[B33] Vingtdeux V. Hamdane M. Loyens A. Gelé P. Drobeck H. Bégard S.. (2007). Alkalizing drugs induce accumulation of amyloid precursor protein by-products in luminal vesicles of multivesicular bodies. J. Biol. Chem. 282, 18197–18205. 10.1074/jbc.M609475200 17468104

[B34] Xiao T. Zhang W. Jiao B. Pan C. Z. Liu X. Shen L. (2017). The role of exosomes in the pathogenesis of Alzheimer' disease. Transl. Neurodegener. 6:3. 10.1186/s40035-017-0072-x 28184302 PMC5289036

[B35] Yamamoto N. Matsubara T. Sato T. Yanagisawa K. (2008). Age-dependent high-density clustering of gm1 ganglioside at presynaptic neuritic terminals promotes amyloid β-protein fibrillogenesis. Biochim. Biophys. Acta 1778, 2717–2726. 10.1016/j.bbamem.2008.07.028 18727916

[B36] Yanagisawa K. Odaka A. Suzuki N. Ihara Y. (1995). GM1 ganglioside-bound amyloid beta-protein (a Beta): a possible form of preamyloid in Alzheimer's disease. Nat. Med. 1, 1062–1066. 7489364 10.1038/nm1095-1062

[B37] Yuyama K. Igarashi Y. (2016). Physiological and pathological roles of exosomes in the nervous system. Biomol. Concepts 7, 53–68. 10.1515/bmc-2015-0033 26812803

[B38] Yuyama K. Sun H. Mitsutake S. Igarashi Y. (2012). Sphingolipid-modulated exosome secretion promotes clearance of amyloid-β by microglia. J. Biol. Chem. 287, 10977–10989. 10.1074/jbc.M111.324616 22303002 PMC3322859

[B39] Yuyama K. Sun H. Sakai S. Mitsutake S. Okada M. Hidetoshi T.. (2014). Decreased Amyloid-β pathologies by intracerebral loading of glycosphingolipid-enriched exosomes in Alzheimer model mice. J. Biol. Chem. 289, 24488–24498. 10.1074/jbc.M114.577213 25037226 PMC4148874

[B40] Yuyama K. Sun H. Usuki S. Sakai S. Hanamatsu H. Mioka T.. (2015). A Potential function for neuronal exosomes: sequestering intracerebral amyloid-β peptide. FEBS Lett. 589, 84–88. 10.1016/j.febslet.2014.11.027 25436414

[B41] Zhuang X. Xiang X. Grizzle W. Sun D. Zhang S. Axtell R. C.. (2011). Treatment of brain inflammatory diseases by delivering exosome encapsulated anti-inflammatory drugs from the nasal region to the brain. Mol. Ther. 19, 1769–1779. 10.1038/mt.2011.164 21915101 PMC3188748

